# Reliability and Validation of the Child Eating Behavior Questionnaire in 3- to 6-Year-Old Spanish Children

**DOI:** 10.3389/fpsyg.2022.705912

**Published:** 2022-05-04

**Authors:** Andrea Jimeno-Martínez, Ivie Maneschy, Luis A. Moreno, Gloria Bueno-Lozano, Pilar De Miguel-Etayo, Katherine Flores-Rojas, Jose Manuel Jurado-Castro, Carmela de Lamas, Rocio Vázquez-Cobela, Raúl Martinez-Lacruz, Olga Portoles, J. Alfredo Martínez, Santiago Navas-Carretero, Helmut Schröder, Montserrat Fitó, Nancy Babio, Jordi Salas-Salvadó, Rosaura Leis, Mercedes Gil-Campos, Azahara I. Rupérez

**Affiliations:** ^1^Growth, Exercise, NUtrition and Development (GENUD) Research Group, Facultad de Ciencias de la Salud, Universidad de Zaragoza, Instituto Agroalimentario de Aragón (IA2) and Instituto de Investigación Sanitaria de Aragón (IIS Aragón), Zaragoza, Spain; ^2^Consorcio CIBER, M.P. Fisiopatología de la Obesidad y Nutrición (CIBEROBN), Instituto de Salud Carlos III (ISCIII), Madrid, Spain; ^3^Metabolism Unit, Maimonides Biomedical Research Institute of Cordoba (IMIBIC), Reina Sofia University Hospital, University of Córdoba, Córdoba, Spain; ^4^Escuela Universitaria de Osuna (Centro Adscrito a la Universidad de Sevilla), Osuna, Spain; ^5^Pediatric Nutrition Research Group, Health Research Institute of Santiago de Compostela (IDIS), Santiago de Compostela, Spain; ^6^Unit of Investigation in Human Nutrition, Growth and Development of Galicia (GALINUT), University of Santiago de Compostela, Santiago de Compostela, Spain; ^7^Department of Preventive Medicine, University of Valencia, Valencia, Spain; ^8^Department of Nutrition, Food Sciences and Physiology, Center for NutritionResearch, University of Navarra, Pamplona, Spain; ^9^Precision nutrition programs, IMDEA Research Institute on Food and Health Sciences, Madrid, Spain; ^10^Centro de Investigación Biomédica en Red Epidemiología y Salud Pública (CIBERESP), Instituto de Salud Carlos III, Madrid, Spain; ^11^Unit of Cardiovascular Risk and Nutrition, Hospital del Mar, Institut Municipald’Investigació Médica (IMIM), Barcelona, Spain; ^12^Universitat Rovira i Virgili, Departament de Bioquímica i Biotecnologia, Unitat de Nutrició Humana, Reus, Spain; ^13^Institut d’Investigació Sanitària Pere Virgili (IISPV), University Hospital of Sant Joan de Reus, Reus, Spain; ^14^Unit of Pediatric Gastroenterology, Hepatology and Nutrition, Pediatric Service, University Clinical Hospital of Santiago (CHUS), Santiago de Compostela, Spain

**Keywords:** eating behavior, childhood obesity, body mass index, child eating behavior questionnaire, validation, reliability

## Abstract

**Introduction:**

Eating behavior is often established during the first years of life. Therefore, it is important to make a research on it to understand the relationships that children have with food and how this can contribute to prevent the development of childhood obesity. An appropriate assessment of eating behavior can be achieved using the “Child Eating Behavior Questionnaire” (CEBQ). This questionnaire has been validated in several populations and languages, but it has never been translated, adapted, and validated for Spanish children.

**Aim:**

To evaluate the reliability and internal consistency of the CEBQ questionnaire, culturally adapted and translated into Spanish (Spain), in Spanish families with children aged 3 to 6 years, as well as its association with children’s body mass index (BMI) to test its construct validity.

**Materials and Methods:**

Children between 3 and 6 years old were recruited from the ongoing MELI-POP randomized controlled clinical trial, as well as from public schools located in middle class neighborhoods of Zaragoza, Spain, to complete the sample. Sociodemographic characteristics and anthropometric measures were obtained according to standardized methods. The 35-item CEBQ questionnaire was completed twice with a time difference of 3 weeks between each response. Statistical analyses included the evaluation of internal consistency and reliability of the questionnaire, a confirmatory factor analysis, and the association between the different CEBQ scales and the children’s BMI.

**Results:**

A total of 197 children completed variables; 97 of them were boys (49.2%) and 100 girls (50.8%). Mean age of the total sample was 4.7 ± 0.9 years. There was a high test-re-test reliability of the questionnaire with values close to 1, with an average of 0.66 and a good internal consistency (Cronbach alpha with values above 0.7), so that a high reliability is established between the items in each scale. A gradual positive association was found between the score of different “pro-intake” scales of the CEBQ: “Food Responsiveness,” “Emotional Overeating,” and “Enjoyment of food” and the children’s BMI; at the opposite, negative associations were observed between BMI and the score of anti-intake scales “Satiety Responsiveness,” “Slowness in Eating,” and “Emotional Undereating.”

**Conclusion:**

The Spanish version of the CEBQ is a useful tool to assess the eating behavior of Spanish children because the high reliability and internal validity. There is a significant association between eating behavior and BMI in Spanish children.

## Introduction

Childhood obesity has become a major public health problem in every country in the world. The problem is global and is progressively affecting many low- and middle-income countries, especially in urban areas. Average BMI and obesity prevalence increased worldwide in children and adolescents from 1975 to 2016, with the rate of change in average BMI moderately correlated with that of adults until around 2000, but weakly correlated thereafter. The trend in average BMI for children and adolescents has stabilized, albeit at elevated levels, in many high-income countries since around 2000, but has accelerated in many other countries. If post-2000 trends continue, childhood and adolescent obesity are expected to overtake moderate and severe underweight by 2022 (Bentham et al., 2017). Prevalence has increased at an alarming rate. It is estimated that in 2016, more than 41 million children under 5 years of age worldwide had overweight or obesity. In recent years, the prevalence of childhood obesity in Europe has increased (World Health Organization. Childhood Obesity Surveillance Initiative, 2017). Particularly, in Spain, 23.2% of children between 6 and 9 years old have overweight and 18.1% have obesity, being these among the highest prevalence in Europe (Ortega Anta et al., 2016).

Obesity in children is mainly due to a positive energy balance, with an excessive energy intake and/or a low energy expenditure, combined with a genetic predisposition for weight gain (Moreno et al., 2008). However, most children with obesity do not have a unique genetic or underlying endocrine cause for their weight gain (Kumar and Kelly, 2017), which points to lifestyle as the main drivers of early obesity development.

Among lifestyle factors, eating habits and food preferences are of great importance (Todendi et al., 2020). These behavioral traits are acquired in early childhood and may change over time according to individual experiences. If these habits are adequate, they will contribute to guarantee health in adulthood (Qorbani et al., 2020). Children’s eating habits are influenced by the characteristics of their parents and family, including their origin and educational level, among others (Iguacel et al., 2018).

There is a wide variety of tools that have been used to assess eating behavior. Traditionally, the study of the factors that influence food consumption has been based on the measurement of intake using 24-h recalls or food frequency questionnaires. Most nutritional surveys, especially in the adult population, have used these methods, in combination with others such as the diet history (Castell et al., 2015). However, in recent years, questionnaires have also been developed and used to measure eating behavior in children. Some examples are the “Toddler Feeding Style Questionnaire” (TFSQ) (Avecilla-benítez et al., 2019), the “Children Feeding Questionnaire” (CFQ) (Ek et al., 2016), the “Dutch Eating Behavior Questionnaire” adapted to children (DEBQ-C) (Baños et al., 2011), the “Child Three-Factor Eating Questionnaire” (CTFEQr17, (Bryant et al., 2018), and the Child Eating Behavior Questionnaire (CEBQ) (Wardle et al., 2001). Indeed, these psychometric tools have been used to assess eating behavior in children and adults in order to predict the risk of eating disorders and body weight-related problems. Eating behavior scores obtained from questionnaires in children represent subjective information that may change over time. However, they have advantages over dietary intake reports in that they can be answered by an informant (usually the mother), who has almost complete observational access to their children in a wide range of situations (Wardle et al., 2001).

Currently, the CEBQ is considered one of the most comprehensive instruments for assessing children’s eating behavior (Sleddens et al., 2008). This 35-item questionnaire was developed by Wardle et al. in the United Kingdom (Wardle et al., 2001), to assess children’s eating styles in terms of their association to obesity. The CEBQ groups the items in a total of eight scales: four pro-intake [Food Responsiveness (FR), Enjoyment of Food (EF), Emotional Overeating (EOE), and Desire to Drink (DD)] and four anti-intake (Satiety Responsiveness (SR), Slowness in Eating (SE), Emotional Undereating (EUE), and Food Fussiness (FF)) (Wardle et al., 2001). Since the validation of this questionnaire in 2007 (Carnell and Wardle, 2007), it has been used in many different studies and validated in other populations such as low-income preschool children in the United States (Domoff et al., 2016), three ethnically diverse Australian samples (Battistutta et al., 2013), in a multi-ethnic Asian population (Quah et al., 2017), in Swedish preschoolers (Svensson et al., 2011) or Portuguese children (Viana et al., 2008). For doing some of these validations, CEBQ has been translated into different languages, including a Chilean-Spanish version (González and Martínez, 2011; Santos et al., 2011). However, it has not yet been translated into Spanish (Spain) or validated in Spain against obesity or its related factors.

The aim of this study is to evaluate the reliability and internal consistency of the CEBQ questionnaire, culturally adapted and translated into Spanish (Spain), and to carry out its construct validation in relation with obesity measures in Spanish children aged three to 6 years. The hypothesis of this study is that both the internal consistency and reliability of the Child Eating Behavior Questionnaire translated into Spanish and culturally adapted will be high. In addition, a high association between unhealthy eating habits and the prevalence of childhood obesity is expected. The latter will be analyzed with construct validity as previously done in other languages (Carnell and Wardle, 2007; Sleddens et al., 2008; Viana et al., 2008; Santos et al., 2011; Svensson et al., 2011; Battistutta et al., 2013; Domoff et al., 2016; Quah et al., 2017).

## Materials and Methods

### Design

The validation of this questionnaire is included within the framework of the MELI-POP (Mediterranean Lifestyle in Pediatric Obesity Prevention) pilot study. This study is a multicenter, parallel, randomized, and controlled clinical trial performed in a cohort of children from 3 to 6 years old and at risk of obesity, assessing whether an intervention during childhood, based on the promotion of a Mediterranean eating pattern and regular physical activity, compared to a control group, decreases the incidence of obesity, with a planned follow-up of 10 years. This study is registered in *Clinical trials* with the reference number: NCT04597281.

### Participants

According to previous studies, the sample size in this type of analysis depends on the variables studied, the indicators and the loading factor. It was established as appropriate to carry out the present study with a minimum of 150–200 individuals (Wolf et al., 2013). A total of 204 participants were recruited. From this sample, 100 participants were recruited in primary healthcare centers from the ongoing MELI-POP study in the following cities: 38 participants from Zaragoza, 26 from Córdoba, 21 from Santiago de Compostela, 8 from Reus, and 7 from Valencia, as well as the others 104 from public schools located in middle class neighborhoods of Zaragoza, Spain. Inclusion criteria were as: children whose main language at home included Castilian Spanish, aged 3 to 6 years that participated in the MELI-POP study or attended any of the selected schools. Exclusion criteria were as: age outside the age range of 3 to 6 years, children whose home language was not Spanish and children whose both parents were of non-Spanish origin and perhaps may misunderstand the questionnaire. From the 204 initial participants, after excluding those not meeting the study requirements, data from 197 children were available.

The study was approved by the Clinical Research Ethics Committee of Aragon, Spain, in accordance with national regulations, and the families of infants were informed about the study before giving their written informed consent.

### Procedures

Once the consents were collected, a first visit was made to carry out the anthropometric measurements and to deliver a general questionnaire and the CEBQ to the parents, who would return them in the next visit once completed. Then, the CEBQ was delivered a second time after 21 days, to be filled out by the same caregiver as in the first delivery. This time was chosen as long enough to allow the effects of memory to fade and prevent fatigue, but not so long as to allow lifestyle changes to occur that might affect reliability. These questionnaires were completed prior to the start of the intervention.

### Sociodemographic Characteristics

The demographic characteristics of the participants and information regarding age, sex, date of birth, race, language spoken at home, child’s and parent’s place of birth and parental education, professional qualifications, and occupation were gathered through a general questionnaire.

### Spanish (Spain) Translation and Cultural Adaptation of the CEBQ

The translation of the questionnaire was performed following available recommendations (Tsang and Royse, 2017). A direct and reverse translation was made as in the study by González et al. *(*González and Martínez, 2011*)*. First, the CEBQ was translated from original English into Spanish by two independent translators. In case of translation differences between translators, the best option was agreed upon based on clarity and meaning of the sentence. Then, a reverse translation (from Spanish to English) was made of the version obtained in the first translation by another independent translator, to ensure its accuracy. The understanding of the questionnaire in Spanish was then assessed along with 10 mother–child duos. They were questioned about words that were difficult to understand or statements that were not very precise so that they could propose others that were more natural and understandable. A thorough assessment was made of possible items formulated in reverse, as they could lead to a biased response. After this, the questionnaire was adapted based on the above and concluded with the Spanish (Spain) version of the CEBQ items found in Supplementary Materials 1

### Child Eating Behavior Questionnaire

The 35 items of the CEBQ questionnaire were completed by the primary caregiver twice (Wardle et al., 2001). Each item was to be answered on a Likert-type scale with possible scores from 1 to 5, where 1 is complete absence (never) and 5 is the highest intensity of specific eating behavior (always).

### Anthropometric Measurements

A team of trained professionals measured the weight (kg) and height (m) of the children according to standardized methods. For this purpose, volunteers had to be barefoot and in underwear. The SECA 213 stadiometer (SECA, Hamburg, Germany) was used to determine height (minimum measuring range 60 to 220 cm and accuracy of 0.1 cm). Regarding weight, an electronic balance (SECA 813, SECA, Hamburg, Germany) was used. From these data, body mass index (BMI) was calculated, and children were classified according to their age and sex as having underweight, normal weight, overweight, or obesity, using the cut-off points of Cole and Lobstein (2012). The BMI z-score was calculated based on Spanish reference values according to the child’s sex and age (Sobradillo et al., 2000).

### Statistical Analyses

The general characteristics of the study sample were analyzed by the mean and standard deviation in continuous variables such as the CEBQ items and children’s age, weight, and height, and by N and proportion (%) of the sample for categorical variables such as sex and BMI categories.

#### Evaluation of Internal Consistency and Reliability

The internal consistency of the questionnaire was evaluated of each item over time, reflecting the extent to which the interviewees understand the items of the CEBQ. Once those questionnaires that had not been completed by the same person on both occasions were removed from the sample, the test re-test reliability was also evaluated by means of the intraclass correlation coefficient (ICC), a Pearson’s correlation modality, to check the extent to which the responses of individuals to the items of the questionnaire remained relatively consistent in the repeated administration of the questionnaire. A higher stability coefficient indicated greater reliability of the questionnaire, and errors are not due to changes in individual responses. For the intraclass correlation coefficients, the “single measure” has been chosen because it is the calculation of each individual item and not the set of all items in the questionnaire (which would be measured as an average). This single measure has also been chosen since it is established when the estimator that has answered both questionnaires is the same and it is desired to evaluate the variability in their responses.

#### Confirmatory Factor Analysis

Prior to further statistical analysis, negative items were reversed by recoding (values of 1 were set to 5, values of 2 were set to 4, values of 3 were unchanged, values of 4 were set to 2, and values of 5 were set to 1).

Next, the number of factors in the questionnaire and their loading factor were analyzed to see if they corresponded to those already established by the original authors. For this purpose, Confirmatory factor analyses (CFA) were performed on the original 8-factor model (Sparks and Radnitz, 2012).

For each CFA, based on previous studies (Battistutta et al., 2013; Domoff et al., 2016; Quah et al., 2017), the factor variance was set at 1 and intercorrelations between each of the factors were allowed. The errors are also kept uncorrelated in these analyses and no cross-factor loads are allowed, as recommended in the literature (Hu and Bentler, 1999). The eight factors with eigen values greater than 1 that explain 73% of the total variance were identified using the maximum similarity method after having performed the parallel analysis. The Varimax rotation was then performed, to obtain the rotated solution and to be able to classify each item according to the highest value on its corresponding scale. Model fit was assessed using the root mean square error of approximation (RMSEA), as it has been done in previous papers (Domoff et al., 2016). In addition to these fit indices, factor loadings, squared mean residuals, and modification index were examined to establish model fit.

Next, the items were classified in the scales according to their loading factor and new variables for each scale were calculated with the mean values of their corresponding items. Then, an internal consistency reliability analysis was performed for each scale. The Cronbach alpha value was calculated to observe to what extent the items in the questionnaire are interrelated within each scale, or whether they consistently measure the same parameter. Cronbach alpha = 0 indicated no internal consistency, while alpha = 1 reflected perfect internal consistency.

#### Scales Correlation

Afterward, a bivariate Pearson correlation analysis between the different scales was performed in order to evaluate how the scales were related to each other.

#### Validity Evaluation

Finally, partial correlations between each of the 8 CEBQ scales and the children’s BMI adjusted for sex and age, as well as with the BMI z-score were calculated to analyze the construct validity of the questionnaire.

#### Analysis of CEBQ Scales by Factor

Once the scales were already factored with the corresponding items, the relationship between the different factors and the child’s and parents’ characteristics was evaluated as a test of external validity. As CEBQ scales and behavioral measures were roughly normally distributed, independent t-tests were used to test for sex and age differences in scores and general linear models adjusted for sex and age were used to compare scales among child, maternal, and paternal weight status categories.

SPSS STATISTICS v.26 (IBM Corp. Released 2017) statistical software was used to perform all statistical analysis. The value *p* < 0.05 was established as indicative of significant findings.

## Results

### General Characteristics of the Study Population

The general characteristics of the sample are described in Table 1. There were no significant differences in any of these variables. As there were no children with obesity, BMI status was categorized as underweight, normal weight, and overweight.

**Table 1:** General characteristics of participants.

	Total	Boys	Girls	*P*
Sex	197(100%)	97(49.2%)	100(50.8%)	
Age (y.o)	4.7 ± 0.9	4.7 ± 0.9	4.6 ± 0.9	0.829
Weight (kg)	18.6 ± 3.5	18.9 ± 3.6	18.4 ± 3.3	0.242
Height (cm)	107.1 ± 7.7	108.1 ± 8.2	106.2 ± 7.0	0.122
BMI status				0.123
Underweight	17(8.6%)	7(7.2%)	10(10%)	
Normal weight	145(73.6%)	77(79.4%)	68(68%)	
Overweight	35(17.8%)	13(13.4%)	22(22%)	

*The data are shown as mean ± standard deviation for continuous variables and N (%) for categorical variables. p: significance in the student t-test for numerical variables (age, weight, and height) or the Chi-square test for categorical variables (BMI status)*.

### Internal Consistency and Reliability of the CEBQ

A generally high internal consistency and re-test reliability of the questionnaire’s items were obtained, as shown in Table 2.

**Table 2:** Reliability analysis of the individual items of the children eating behavior questionnaire.

Item	N (%)	ICC	95% CI		*p*
			Lower limit	Upper limit	
My child loves food	183 (92.9%)	0.711	0.632	0.776	<0.001
My child eats more when worried	181 (91.9%)	0.426	0.297	0.540	<0.001
My child has a big appetite	183 (92.9%)	0.744	0.671	0.803	<0.001
My child finishes his/her meal quickly	183 (92.9%)	0.676	0.589	0.747	<0.001
My child is interested in food	183 (92.9%)	0.660	0.569	0.735	<0.001
My child is always asking for a drink	180 (91.4%)	0.654	0.562	0.730	<0.001
My child refuses new foods at first	184 (93.4%)	0.729	0.649	0.792	<0.001
My child eats slowly	184 (93.4%)	0.705	0.625	0.771	<0.001
My child eats less when angry	183 (92.9%)	0.640	0.539	0.721	<0.001
My child enjoys tasting new foods	184 (93.4%)	0.710	0.630	0.775	<0.001
My child eats less when s/he is tired	184 (93.4%)	0.515	0.376	0.626	<0.001
My child is always asking for food	183 (92.9%)	0.734	0.660	0.795	<0.001
My child eats more when annoyed	183 (92.9%)	0.307	0.172	0.432	<0.001
If allowed to, my child would eat too much	184 (93.4%)	0.725	0.648	0.787	<0.001
My child eats more when anxious	184 (93.4%)	0.521	0.408	0.619	<0.001
My child enjoys a wide variety of foods	182 (92.4%)	0.698	0.615	0.765	<0.001
My child leaves food on his/her plate at the end of a meal	184 (93.4%)	0.702	0.621	0.769	<0.001
My child takes more than 30 min to finish a meal	182 (92.4%)	0.783	0.719	0.833	<0.001
Given the choice, my child would eat most of the time	184 (93.4%)	0.757	0.684	0.814	<0.001
My child looks forward to mealtimes	183 (92.9%)	0.691	0.607	0.760	<0.001
My child gets full before his/her meal is finished	184 (93.4%)	0.561	0.454	0.653	<0.001
My child enjoys eating	183 (92.9%)	0.775	0.710	0.827	<0.001
My child eats more when she is happy	183 (92.9%)	0.589	0.486	0.676	<0.001
My child is difficult to please with meals	184 (93.4%)	0.697	0.615	0.764	<0.001
My child eats less when upset	183 (92.9%)	0.647	0.554	0.724	<0.001
My child gets full up easily	183 (92.9%)	0.678	0.591	0.749	<0.001
My child eats more when s/he has nothing else to do	183 (92.9%)	0.655	0.546	0.731	<0.001
Even if my child is full up s/he finds room to eat his/her favorite food	184 (93.4%)	0.724	0.647	0.786	<0.001
If given the chance, my child would drink continuously throughout the day	184 (93.4%)	0.629	0.532	0.709	<0.001
My child cannot eat a meal if s/he has had a snack just before	184 (93.4%)	0.560	0.452	0.652	<0.001
If given the chance, my child would always be having a drink	181 (91.9%)	0.654	0.562	0.730	<0.001
My child is interested in tasting food s/he has not tasted before	182 (92.4%)	0.697	0.613	0.764	<0.001
My child decides that s/he does not like a food, even without tasting it	184 (93.4%)	0.752	0.682	0.809	<0.001
If given the chance, my child would always have food in his/her mouth	183 (92.9%)	0.654	0.563	0.730	<0.001
My child eats more and more slowly during the course of a meal	184 (93.4%)	0.569	0.463	0.660	<0.001

*The data are shown N (%) for categorical variables. Reliability of the CEBQ has been calculated with the intraclass correlation coefficient (ICC). The confidence interval (CI) is 95% and the lower and upper limits are shown*.

Regarding the test re-test reliability, which indicates whether individuals’ responses to the questionnaire items, remain relatively consistent across the same questionnaire administered repeatedly, having most items an ICC above 0.600. As in the case of internal consistency, item 13 stands out with an ICC of 0.307, which could imply differences in the responses to that question between the first and second questionnaire deliveries. However, the item was decided not to be removed in order to maintain the original set of items. It should be noted that in the re-test reliability analysis, N is between 180 and 184 because some of the parent’s participants did not answer all the questions in one or in the two administrations of the questionnaire.

### Confirmatory Factor Analysis

After ensuring that a high internal consistency and adequate re-test reliability were obtained, the confirmatory analysis was performed. Its results are shown in Table 3, where each scale groups the related items according to their factor loading.

**Table 3:** Loading factors for children eating behavior questionnaire items estimated from confirmatory factor analysis.

Factor	Variance %	Scale	Factor loading	Cronbach alpha	MacDonald’s ω
Factor 1	25.8%	Food fussiness		0.896	0.892
		7. My child refuses new foods at first	0.867		
		10. My child enjoys tasting new foods	0.898		
		16. My child enjoys a wide variety of foods	0.666		
		24. My child is difficult to please with meals	0.585		
		32. My child is interested in tasting food s/he has not tasted before	0.899		
		33. My child decides that s/he does not like a food, even without tasting it	0.727		
Factor 2	15.4%	Food responsiveness		0.865	0.865
		12. My child is always asking for food	0.697		
		14. If allowed to, my child would eat too much	0.702		
		19. Given the choice, my child would eat most of the time	0.827		
		28. Even if my child is full up s/he finds room to eat his/her favorite food	0.720		
		34. If given the chance, my child would always have food in his/her mouth	0.760		
Factor 3	8.4%	Satiety responsiveness		0.803	0.808
		3. My child has a big appetite (−0.579 in Factor 5)	0.320		
		17. My child leaves food on his/her plate at the end of a meal	0.704		
		21. My child gets full before his/her meal is finished	0.734		
		26. My child gets full up easily	0.748		
		30. My child cannot eat a meal if s/he has had a snack just before	0.678		
Factor 4	6.9%	Emotional undereating		0.829	0.842
		9. My child eats less when angry	0.836		
		11. My child eats less when s/he is tired	0.696		
		23. My child eats more when she is happy	0.778		
		25. My child eats less when upset	0.846		
Factor 5	4.3%	Enjoyment of food		0.839	0.846
		1. My child loves food	0.678		
		5. My child is interested in food (−0.554 in Factor 1)	0.369		
		20. My child looks forward to mealtimes	0.492		
		22. My child enjoys eating	0.578		
Factor 6	3.9%	Desire to drink		0.878	0.895
		6. My child is always asking for a drink	0.840		
		29. If given the chance, my child would drink continuously throughout the day	0.884		
		31. If given the chance, my child would always be having a drink	0.906		
Factor 7	3.7%	Slowness in eating		0.777	0.774
		4. My child finishes his/her meal quickly	0.656		
		8. My child eats slowly	0.658		
		18. My child takes more than 30 min to finish a meal	0.784		
		35. My child eats more and more slowly during the course of a meal	0.687		
Factor 8	2.9%	Emotional overeating		0.746	0.761
		2. My child eats more when worried	0.684		
		13. My child eats more when annoyed	0.785		
		15. My child eats more when anxious	0.768		
		27. My child eats more when s/he has nothing else to do (0.668 in Factor 2)	0.357		

*Confirmatory factor analysis based on the loading factor and items grouped by scale. The internal consistency of the scales was calculated with the Cronbach alpha and the MacDonald’s ω. Additional factor loadings for items that loaded in more than one scale are indicated*.

The eight expected scales: Food Responsiveness (FR), Enjoyment of Food (EF), Emotional Overeating (EOE), Desire to Drink (DD), Satiety Responsiveness (SR), Slowness in Eating (SE), Emotional Undereating (EUE), and Food Fussiness (FF), were identified in the unforced model. The 8 factor model was found to have reasonable fit to the data. The 35 elements loaded in the respective eight factors (with factor loadings above 0.30, *p* < 0.001) and the mean squared residuals after varimax rotation were above 1. Table 3 is ordered according to the decreasing percentage of variance explained in the factor analysis of the main component.

Most items were grouped into the expected factor with a few exceptions. Item 5 was grouped in two scales, showing a lower loading factor in its corresponding scale “Enjoyment of food” and a higher (but negative) one in the “Food Fussiness” scale. Another cross load appears in item 27, which is supposed to belong to the “Emotional Overeating” scale but showed a high loading factor in the “Food Responsiveness” scale with a value of 0.668 compared to 0.357 on its original scale. It is also worth noting the results with respect to item 3. This is a reverse-scored item, which was originally categorized in the “Satiety Responsiveness” scale, that showed a low loading factor (0.320) on this scale. However, it shows a higher negative loading factor of −0.579 on the “Food Responsiveness” scale (the negative value due to being a reverse-scored item). For the rest of the analyses, the load corresponding to its original scale was used.

Concerning the reliability between the scales, after calculating Cronbach’s alpha, all the values are above 0.7, so that a high reliability is established between the items in each scale. The same is true for MacDonald’s ω, which has very similar values to the previous one, verifying that the internal consistency is high.

As for the % variance, the first factors explain relatively large amounts of variance (especially factor 1, “Food fussiness”), while the subsequent factors explain smaller amounts of variance.

### Correlation Between CEBQ Scales

Table 4 shows the results of the Pearson’s correlation analysis between the different scales, with many statistically significant correlations. The strongest positive correlation was between the “Emotional Overeating” and “Food Responsiveness” scales. Similarly, “Satiety Responsiveness” and “Slowness in Eating” scales, as well as “Food Responsiveness” and “Enjoyment of food” were positively correlated. On the other hand, several negative correlations were found between opposite scales, highlighting the ones between “Enjoyment of food” and “Satiety responsiveness” and “Slowness in eating.”

**Table 4:** Correlation between the scores for the different scales of the Children Eating Behavior Questionnaire.

	FR	EOE	EF	DD	SR	SE	EUE	FF
Food Responsiveness (FR)	1							
Emotional Overeating (EOE)	0.642^**^	1						
Enjoyment of food (EF)	0.516^**^	0.198^**^	1					
Desire to Drink (DD)	0.236^**^	0.203^**^	0.004	1				
Satiety Responsiveness (SR)	−0.400^**^	−0.117	−0.679^**^	0.085	1			
Slowness in Eating (SE)	−0.387^**^	−0.233^**^	−0.513^**^	0.080	0.557^**^	1		
Emotional Undereating (EUE)	0.024	0.206^**^	−0.143^*^	0.100	0.328^**^	0.237^**^	1	
Food fussiness (FF)	−0.115	0.034	−0.611^**^	0.122	0.417^**^	0.302^**^	0.181^*^	1

*Pearson’s correlation analysis between the different scales*.

* *p < 0.05*;

** *p < 0.001*.

### Validity of the Questionnaire. Analysis of the Association Between the Scales, Age-, and Sex-Adjusted BMI and BMI z-Score

The results from the partial correlation analyses between BMI and the CEBQ scales of the participants, adjusted for age and sex and with BMI z-score, are shown in Table 5. Significant positive associations were found between BMI and the pro-intake questionnaire scales “Food Responsiveness,” “Emotional Overeating,” and “Enjoyment of food.” There was a statistically significant correlation that means that these scales in the questionnaire have a correlation with the child’s BMI.

**Table 5:** Construct validity of the children eating behavior questionnaire. Correlation between scales and children’s BMI.

	*N*	Partial correlation with BMI (sex, age)	*p*	Correlation with BMI Z-score	*p*
Food responsiveness (FR)	197	0.297	<0.001	0.268	<0.001^**^
Emotional overeating (EOE)	197	0.187	0.009	0.160	0.025^*^
Enjoyment of food (EF)	197	0.179	0.012	0.186	0.009^*^
Desire to drink (DD)	197	0.134	0.061	0.101	0.157
Satiety responsiveness (SR)	197	−0.283	<0.001	−0.318	<0.001^**^
Slowness in eating (SE)	197	−0.276	<0.001	−0.291	<0.001^**^
Emotional undereating (EUE)	197	−0.092	0.201	−0.168	0.018^*^
Food fussiness (FF)	197	−0.007	0.922	−0.006	0.937

*Correlation between CEBQ scales and body mass index adjusted for age and sex, and between CEBQ scales and Body Mass Index (BMI) Z-score*.

* *p < 0.05;*

** *p < 0.001*.

On the other hand, the anti-intake scales “Satiety Responsiveness” and “Slowness in Eating” were negatively associated with BMI. The correlation with BMI z-score also was significant in “Emotional Undereating.”

### Analysis of CEBQ Scales by Factor

Significant differences were observed between boys and girls in the scales “Emotional Overeating” that showed a higher score in girls, and “Slowness in Eating,” with a higher score in boys (Table 6). No differences were observed in the different CEBQ scales according to age (data not shown).

**Table 6:** External validity of children eating behavior questionnaire.

	Boys		Girls		*p*
	*N*	Mean (SD)	*N*	Mean	
Food responsiveness (FR)	97	2.03(0.77)	100	2.24(0.92)	<0.001^**^
Emotional overeating (EOE)	97	1.62_a_(0.53)	100	1.86_b_(0.67)	<0.050^*^
Enjoyment of food (EF)	97	3.27(0.79)	100	3.36 (0.78)	<0.001^**^
Desire to Drink (DD)	97	2.32(0.93)	100	2.34 (0.91)	<0.001^**^
Satiety responsiveness (SR)	97	2.95(0.77)	100	2.90(0.72)	<0.001^**^
Slowness in eating (SE)	97	3.20_a_(0.89)	100	2.94_b_(0.76)	<0.050^*^
Emotional undereating (EUE)	97	3.16(1.01)	100	3.16(0.90)	<0.001^**^
Food fussiness (FF)	97	2.80(0.93)	100	2.94(0.86)	<0.001^**^

*Analysis of the scales by child’s sex. Different subscript letters indicate significant differences in the pairwise comparison*.

* *p < 0.05;*

** *p < 0.001*.

Significant differences were also observed between children’s weight status and their scores on the different CEBQ scales (Table 7). Children with overweight showed higher values than those with normal weight and underweight in the “Food Responsiveness” scale while they had lower values on the “Satiety Responsiveness” and “Slowness in eating” scales.

**Table 7:** Mean values of the children eating behavior questionnaire scales according to children weight status.

		Underweight			Normal weight			Overweight		
Child weight status										*p* (sex. age)
	*N*	Mean	SD	*N*	Mean	SD	*N*	Mean	SD	
Food responsiveness (FR)	17	1.59_a_	0.43	145	2.10_b_	0.83	35	2.53_c_	0.94	0.001^*^
Emotional overeating (EOE)	17	1.46_a_	0.44	145	1.75_a_	0.64	35	1.84_a_	0.58	0.086
Enjoyment of food (EF)	17	3.04_a_	0.85	145	3.29_a_	0.77	35	3.51_a_	0.82	0.179
Desire to drink (DD)	17	2.08_a_	0.78	145	2.30_a_	0.90	35	2.54_a_	1.05	0.108
Satiety responsiveness (SR)	17	3.28_a_	0.79	145	2.95_a,b_	0.73	35	2.65_b_	0.71	0.030^*^
Slowness in eating (SE)	17	3.74_a_	0.66	145	3.05_b_	0.80	35	2.84_b_	0.92	0.001^*^
Emotional undereating (EUE)	17	3.21_a,b_	0.80	145	3.24_a_	0.92	35	2.79_b_	1.09	0.263
Food fussiness (FF)	17	3.17_a_	0.91	145	2.81_a_	0.86	35	2.97_a_	1.00	0.259

*Scale mean values and standard deviation (SD) according to children weight status. p: general linear model adjusted for sex and age. Different subscript letters indicate significant differences in the pairwise comparison*.

* *p < 0.05*.

As for parental characteristics, significant associations were found between maternal weight status and several CEBQ scales (Table 8). The “Enjoyment of food” scale was negatively associated with maternal obesity, with lower values for children whose mothers had obesity with a significant difference with those with normal weight mothers. Surprisingly, a significant association was also found with “Satiety responsiveness,” in which children born to mothers with overweight or obesity had higher values in this scale. In the case of paternal weight status, no significant associations were observed with any of the CEBQ scales (data not shown).

**Table 8:** Mean values of the children eating behavior questionnaire scales according to maternal weight status.

	Maternal weight status											
Underweight			Normal weight			Overweight			Obesity		
													*p* (sex, age)
	*N*	Mean	SD	*N*	Mean	SD	*N*	Mean	SD	*N*	Mean	SD	
Food responsiveness (FR)	6	1.57_a_	0.34	79	2.23_a_	0.86	33	2.15_a_	0.81	76	2.09_a_	0.90	0.309
Emotional overeating (EOE)	6	1.29_a_	0.25	79	1.86_a_	0.61	33	1.64_a_	0.59	76	1.72_a_	0.63	0.093
Enjoyment of food (EF)	6	3.13_a,b_	0.54	79	3.51_a_	0.79	33	3.33_a,b_	0.90	76	3.12_b_	0.73	0.012^*^
Desire to drink (DD)	6	1.89_a_	0.46	79	2.22_a_	0.82	33	2.35_a_	1.06	76	2.49_a_	0.97	0.110
Satiety responsiveness (SR)	6	3.17_a,b_	0.99	79	2.75_a_	0.69	33	2.94_a.b_	0.85	76	3.08_b_	0.71	0.009^*^
Slowness in eating (SE)	6	3.67_a_	0.90	79	2.92_a_	0.77	33	3.16_a_	0.82	76	3.13_a_	0.89	0.080
Emotional undereating (EUE)	6	3.00_a,b_	0.81	79	3.35_a_	0.89	33	3.19_a,b_	0.99	76	2.93_b_	0.98	0.158
Food fussiness (FF)	6	2.75_a_	0.77	79	2.70_a_	0.78	33	2.88_a_	1.03	76	3.07_a_	0.94	0.055

*Scale mean values and standard deviation (SD) according to maternal weight status. p: general linear model adjusted for sex and age. Different subscript letters indicate significant differences in the pairwise comparison among maternal weight status*.

* *p < 0.05.*

## Discussion

According to our findings, the reliability and internal consistency of the “Children Eating Behavior Questionnaire” translated into Spanish (Spain) and culturally adapted to families with children from 3 to 6 years old has showed to be adequate, as in previous translations to other languages.

Regarding the CFA, a good fit of eight factors in the unforced model was obtained, whereas other authors considered a three-factor model (Battistutta et al., 2013; Domoff et al., 2016). In addition, although several previous studies (Wardle et al., 2001; Carnell and Wardle, 2007; Santos et al., 2011) reported a seven-factor structure, these results indicate an important degree of overlapping between the scales of “Satiety Responsiveness” and “Slowness in eating.” Indeed, it is worth noting the relationship observed between these two scales and the similarities between their respective results in all analyses. This result is also confirmed by the high correlation observed between these two scales, which is maximum compared to the other inter-scale correlations. Regarding the scale-grouped items in the confirmatory factor analysis, those in the “Food fussiness” (factor 1) explain 25.8% of the variance, “Food responsiveness” items (factor 2) explain15.4% of variance, and “Emotional overeating” items (factor 8) explain 2.9% of variance. This means that relatively large amounts of variance are covered by the first factors while the latter contribute quite less. Comparing these results with other studies (Carnell and Wardle, 2007), it can be observed that in our study, the percentages of variance are lower. This is due to the fact that the items that make up this factor have values with a smaller deviation between and with respect to their mean.

As for the grouping of the different scales in this analysis, the results are similar to the existing literature, but it is important to mention the differences with the original eight-factor model structure (Wardle et al., 2001). Concerning item 3 *“My child has a big appetite,”* a reverse-scored item, it was originally grouped in the “Satiety Responsiveness” scale in contrast to our results, where it is grouped in the “Enjoyment of Food” scale with a value of −0.579. Most probably, the reason for this could be that a child with a big appetite may also enjoy food the most. The same cross loading happened in item 5 *“My child is interested in food,”* which shows a lower loading factor in its original scale “Enjoyment of food” and a higher, but inverse one, in the “Food Fussiness” scale. However, the finding is again probably due to the scales being opposite to each other. In relation to item 27 *“My child eats more when s/he has nothing else to do*, it had a higher factor loading on the “Food Responsiveness” scale, although this item is considered to belong to the “Emotional Overeating” scale (Wardle et al., 2001). However, this item has been previously considered within the “Food Responsiveness” in other recent studies (Santos et al., 2011; Domoff et al., 2016). Theoretically, the fact that this item also appears in this scale is consistent with its reflecting external eating, a type of eating behavior that underlies “Food Responsiveness” (Wardle et al., 2001).

Regarding the reliability of the re-test (intra-items), it was not possible to compare it with the existing literature since, unlike in our study, this second delivery of the questionnaire was not carried out previously. We decided to perform this reliability analysis given its methodological usefulness in the validation of questionnaires (Tsang and Royse, 2017), in addition to its obvious importance when assessing the adequacy of a modified questionnaire. The obtained results indicated a high reliability of the questionnaire in time between the answers of the first and second deliveries of the questionnaire.

Although the traits represented by each scale are conceptually different from the other scales, it is obvious that there is a positive relationship between pro- and anti-intake scales as well as inverse correlations between the scales of the two groups. This occurred in a similar way in previous studies (Viana et al., 2008; Battistutta et al., 2013; Quah et al., 2017) and is explained in the study by Carnell et al. (Carnell and Wardle, 2007), where the authors state that pro-intake and anti-intake behaviors could tend to be inherited or learned together, or have a fundamental determinant in common.

The CEBQ has been validated in several languages, but it had never been validated to be used in Spain before. This questionnaire is a useful tool for detecting obesity promoting behaviors and can therefore be helpful in developing effective public health interventions. These results affirm the validity of this questionnaire as a tool for the study of relatively stable eating behavior over time, showing good reliability and high internal consistency, as observed in the existing literature in different populations: The United States (Carnell and Wardle, 2007; Domoff et al., 2016), three ethnically diverse Australian samples (Battistutta et al., 2013), in a multi-ethnic Asian population (Quah et al., 2017), in Swedish preschoolers (Svensson et al., 2011), in Portuguese children (Viana et al., 2008), and even in the Chilean population (Santos et al., 2011).

According to past and present results, eating behavior is clearly associated with overweight and obesity in children. Our results also show a clear and gradual association between CEBQ scores and BMI as reported in previous studies in children with similar ages (Wardle et al., 2001; Viana et al., 2008; Santos et al., 2011). This confirms again the construct validity of the questionnaire in Spanish children, especially in relation to the positive associations between BMI and the food approach (“pro-intake”) scales such as “Enjoyment of Food, “Food Responsiveness,” and “Emotional Overeating.” These results are similar to previous studies (Carnell and Wardle, 2007; Santos et al., 2011; Domoff et al., 2016), including that the subscale “Desire to drink” has not shown difference between the normal weight and overweight participants and no association with obesity has been found. Even so, it is confirmed that children with higher BMI also score higher in the “pro-intake” CEBQ questions and are therefore overly sensitive in their relationship to food. On the other hand, the inverse associations between body weight and the scores of the food avoidance (“anti-intake”) scales such “Satiety Responsiveness” and “Slowness in eating” and “Emotional Undereating” are similar to the above-mentioned studies (Santos et al., 2011; Battistutta et al., 2013; Domoff et al., 2016). As mentioned, in the present study, the scales of “Desire to drink” and “Food Fussiness” related to emotional eating, have not shown any association with obesity.

In the analysis of the CEBQ scales compared by factor, is important to mention the differences that have been found. There were significant differences (*p* < 0.05) regarding sex in two of the scales: “Emotional Overeating” where girls showed higher scores and, surprisingly, “Slowness in Eating” where they showed lower scores. On the contrary, in most studies prior to this one, no significant sex differences in eating behavior have been found except in two studies where differences are found in the “Desire to Drink” scale with higher scores in boys compared to girls (dos Passos et al., 2015; Sanlier et al., 2018). As it has been discussed in other studies, it is important to find the time at which differences appear between boys and girls, as some researchers have suggested that eating behaviors present different characteristics between adolescent by sex, which has been attributed to girls’ self-concern with their body image (Dubois et al., 2007). However, in our study, this reason is not likely due to the young age of the participants.

Concerning children’s weight status and their responses on the CEBQ, our findings are supported by previous studies (Sleddens et al., 2008; Viana et al., 2008; dos Passos et al., 2015; Ayine et al., 2021), whereas the present results point toward children with overweight having a greater interest in food due to their higher scores on the “Food Responsiveness” scale. We also found that children with underweight showed higher scores on the “Satiety Responsiveness” and “Slowness in Eating” scales, which could indicate their lack of interest in food compared to those with higher weight.

Significant associations were also found between maternal weight status and their children’s score in some of the CEBQ scales. Children whose mothers had obesity obtained lower values in the “Enjoyment of food” scale than those with normal weight mothers, but they also obtained higher values in “Satiety Responsiveness.” In addition, an almost significant association was observed for “Food fussiness,” with higher values for children with mothers who had overweight or obesity. Although no studies have been found that relate CEBQ responses with respect to children to parental weight status, other studies have reported that mothers with normal weight are characterized by a higher level of positive eating behaviors compared to mothers with excessive body weight (Pasztak-Opiłka et al., 2020). Another study showed that parental pressure to eat may be associated with child weight through a counterproductive effect of decreasing children’s enjoyment of food. Alternatively, pressure to eat could also be the parental response when children feel “full” quickly. This explanation is supported by the correlation between pressure to eat and satiety responsiveness (Jansen et al., 2012). This supports the results of our study as, possibly, mothers with obesity tend to engage in more negative eating behaviors with their children in an attempt to control their weight. Perhaps the observed findings in the “Enjoyment of Food” scale are because that mothers with obesity exert more pressure on their children because of their concern about weight and this result in children not enjoying food. A similar explanation could be given to children of mothers with obesity having a quicker feeling of satiety as observed through their higher scores on the “Satiety Responsiveness” scale. These results have not been previously found in other studies due to the lack of emphasis on studying the correlation between the CEBQ scales and other external factors such as age and sex of the children, or parental factors such as parental weight status.

Finally, the “Emotional Overeating” scale had the lowest average in association with all the factors analyzed in this study, as observed in previous ones (Wardle et al., 2001; Domoff et al., 2016), as well as a lower internal consistency compared to the rest of the scales. The importance of eating behavior is that it is amenable to modification through appropriate interventions to prevent and/or treat childhood obesity. In this context, changes in CEBQ scores can also be used to assess the effectiveness of such preventive actions (Ford et al., 2010). It is therefore very important that the questionnaire used to measure behavior has been validated with satisfactory results.

Compared to other questionnaires that assess children’s eating behavior, the CEBQ is a suitable option because while other questionnaires such as the DEBQ-C have a 3-scale model, this one has an 8-factor model that measures each behavioral scale more specifically by separating them into pro- and anti-eating scales, which makes for a more specific assessment of eating behavior compared to other less concise models.

To discuss the limitations of this work, it is possible that some of the differences found between this paper and some others are due to the sample size (Battistutta et al., 2013; Domoff et al., 2016; Quah et al., 2017) and cultural different habits of the families. The sample size of the study is not large but is adequate due to the fact that the analysis consists of a 2-factor model with 8 indicators and with loadings on the confirmatory factor analysis (CFA) above 0.65 according to the study by Wolf et al. (2013). About the strengths of this study, it should be noted this work is the first to evaluate the reliability and validity of the CEBQ and its association with the BMI in Spanish children. Furthermore, the repeated delivery of the CEBQ after translation and cultural adaptation, unlike in other studies, makes the validation of the CEBQ appropriate according to the questionnaire validation guide by Tsang and Royse (2017), as this process may generate some error in translation or in understanding the questionnaire before it is carried out. The inclusion of the study of the relationship between the CEBQ scales and parental weight status is a novel aspect that had not been included in previous studies which can be considered in future research.

## Conclusion

Our study supports the original 8-factor structure of the CEBQ translated into Spanish in a sample of Spanish preschool children. This is the first time that the questionnaire has been validated in this language and it has been found to have an adequate reliability. Several scales of the questionnaire showed the expected association with the BMI of the children. The importance of the concept of feeding style lies in its contribution to the understanding of how behavioral pathways relate to obesity. The present results suggest that the CEBQ translated into Spanish is a valuable tool for identifying specific eating behaviors that may be implicated in the development of obesity in children. The use of CEBQ in future research could help us understand inherited behavioral phenotypes and guide obesity prevention interventions.

## Data Availability Statement

The raw data supporting the conclusions of this article will be made available by the authors, without undue reservation.

## Ethics Statement

The studies involving human participants were reviewed and approved by Clinical Research Ethics Committee of Aragon, Spain. Written informed consent to participate in this study was provided by the participants’ legal guardian/next of kin.

## Author Contributions

AR is AJ-M’s co-supervisor. LM is AJ-M’s supervisor. AJ-M, IM, LM, and AR contributed to the conceptualization, methodology, formal analysis, and writing—original draft preparation. AJ-M, IM, PM-E, KF-R, JJ-C, CL, RV-C, RM-L, and NB carried out measurements. AJ-M, IM, LM, PM-E, KF-R, JJ-C, CL, RV-C, RM-L, OP, JM, SN-C, HS, MF, NB, JS-S, RL, MG-C, and AR participated in the writing—review and editing. LM was coordinator of the MELI-POP study, and together with OP, JM, SN-C, HS, MF, NB, JS-S, RL, and MG-C formed the Core Management Group of the project. All authors contributed to the article and approved the submitted version.

## Funding

This study has been funded by Instituto de Salud Carlos III (ISCIII) through the project “PI20/00988” and co-funded by the European Union.

## Conflict of Interest

JS-S is a non-paid member of the Scientific Committee of the International Nut and Dried Fruit Foundation. He has received grants/research support from the American Pistachio Growers and International Nut and Dried Fruit Foundation through his Institution. He is an honorary member of the “Instituto Danone” in Spain and non-paid member of International Danone Institute.

The remaining authors declare that the research was conducted in the absence of any commercial or financial relationships that could be construed as a potential conflict of interest.

## Publisher’s Note

All claims expressed in this article are solely those of the authors and do not necessarily represent those of their affiliated organizations, or those of the publisher, the editors and the reviewers. Any product that may be evaluated in this article, or claim that may be made by its manufacturer, is not guaranteed or endorsed by the publisher.
