# Corrigendum: Reliability and validation of the child eating behavior questionnaire in 3- to 6-year-old Spanish children

**DOI:** 10.3389/fpsyg.2022.1075681

**Published:** 2022-11-15

**Authors:** Andrea Jimeno-Martínez, Ivie Maneschy, Luis A. Moreno, Gloria Bueno-Lozano, Pilar De Miguel-Etayo, Katherine Flores-Rojas, Jose Manuel Jurado-Castro, Carmela de Lamas, Rocio Vázquez-Cobela, Raúl Martinez-Lacruz, Olga Portoles, J. Alfredo Martínez, Santiago Navas-Carretero, Helmut Schröder, Montserrat Fitó, Nancy Babio, Jordi Salas-Salvadó, Rosaura Leis, Mercedes Gil-Campos, Azahara I. Rupérez

**Affiliations:** ^1^Growth, Exercise, NUtrition and Development (GENUD) Research Group, Facultad de Ciencias de la Salud, Universidad de Zaragoza, Instituto Agroalimentario de Aragón (IA2) and Instituto de Investigación Sanitaria de Aragón (IIS Aragón), Zaragoza, Spain; ^2^Consorcio CIBER, M.P. Fisiopatología de la Obesidad y Nutrición (CIBEROBN), Instituto de Salud Carlos III (ISCIII), Madrid, Spain; ^3^Metabolism Unit, Maimonides Biomedical Research Institute of Cordoba (IMIBIC), Reina Sofia University Hospital, University of Córdoba, Córdoba, Spain; ^4^Escuela Universitaria de Osuna (Centro Adscrito a la Universidad de Sevilla), Osuna, Spain; ^5^Pediatric Nutrition Research Group, Health Research Institute of Santiago de Compostela (IDIS), Santiago de Compostela, Spain; ^6^Unit of Investigation in Human Nutrition, Growth and Development of Galicia (GALINUT), University of Santiago de Compostela, Santiago de Compostela, Spain; ^7^Department of Preventive Medicine, University of Valencia, Valencia, Spain; ^8^Department of Nutrition, Food Sciences and Physiology, Center for Nutrition Research, University of Navarra, Pamplona, Spain; ^9^Precision Nutrition Programs, IMDEA Research Institute on Food and Health Sciences, Madrid, Spain; ^10^Centro de Investigación Biomédica en Red Epidemiología y Salud Pública (CIBERESP), Instituto de Salud Carlos III, Madrid, Spain; ^11^Unit of Cardiovascular Risk and Nutrition, Hospital del Mar, Institut Municipald'Investigació Médica (IMIM), Barcelona, Spain; ^12^Universitat Rovira i Virgili, Departament de Bioquímica i Biotecnologia, Unitat de Nutrició Humana, Reus, Spain; ^13^Institut d'Investigació Sanitària Pere Virgili (IISPV), University Hospital of Sant Joan de Reus, Reus, Spain; ^14^Unit of Pediatric Gastroenterology, Hepatology and Nutrition, Pediatric Service, University Clinical Hospital of Santiago (CHUS), Santiago de Compostela, Spain

**Keywords:** eating behavior, childhood obesity, body mass index, child eating behavior questionnaire, validation, reliability

In the published article, the funding information was not provided. The corrected **Funding** statement appears below.

## Funding

This study has been funded by Instituto de Salud Carlos III (ISCIII) through the project “PI20/00988” and co-funded by the European Union.

The authors apologize for this error and state that this does not change the scientific conclusions of the article in any way. The original article has been updated.

## Publisher's note

All claims expressed in this article are solely those of the authors and do not necessarily represent those of their affiliated organizations, or those of the publisher, the editors and the reviewers. Any product that may be evaluated in this article, or claim that may be made by its manufacturer, is not guaranteed or endorsed by the publisher.

