# Supplementary material for: Reliability and Validation of the Child Eating Behavior Questionnaire in 3- to 6-Year-Old Spanish Children
Source: Front Psychol. 2022 May 4;13:705912. doi: 10.3389/fpsyg.2022.705912 (PMC9116151; doi:10.3389/fpsyg.2022.705912)
Supplement: Supplementary file 1 [file Data_Sheet_1.PDF]

Supplementary material 1: Items from CEBQ translated to Spanish

1. A mi hijo/a le encanta la comida
2. Mi hijo/a come más cuando está preocupado
3. Mi hijo/a tiene un gran apetito
4. Mi hijo/a termina su comida muy rápido
5. Mi hijo/a tiene interés en las comidas y los alimentos
6. Mi hijo/a siempre está pidiendo algo de beber
7. Mi hijo/a rechaza los alimentos que no conoce cuando se le ofrecen por primera vez
8. Mi hijo/a come despacio
9. Mi hijo/a come menos cuando está enfadado
10. Mi hijo/a disfruta probando nuevos alimentos
11. Mi hijo/a come menos cuando está cansado
12. Mi hijo/a siempre está pidiendo comida
13. Mi hijo/a come más cuando está molesto o irritado
14. Si se le permitiera, mi hijo/a comería demasiado
15. Mi hijo/a come más cuando está nervioso o inquieto
16. Mi hijo/a disfruta de una gran variedad de alimentos
17. Mi hijo/a deja comida en el plato al final de la comida
18. A mi hijo/a le cuesta más de 30 minutos terminar de comer
19. Si fuera por él/ella, mi hijo/a estaría comiendo la mayoría del tiempo
20. Mi hijo/a espera con ganas las horas de las comidas
21. Mi hijo/a se llena antes de terminar de comer
22. Mi hijo/a disfruta comiendo
23. Mi hijo/a come más cuando está contento/a
24. Es difícil complacer a mi hijo/a con las comidas
25. Mi hijo/a come menos cuando está disgustado
26. Mi hijo/a se llena fácilmente con la comida
27. Mi hijo/a come más cuando no tiene nada que hacer
28. Incluso cuando está lleno, mi hijo/a está dispuesto a comer su comida favorita
29. Si se le diera la oportunidad, mi hijo/a estaría bebiendo continuamente a lo largo de todo el día
30. Mi hijo/a no puede comerse la comida si ha tomado algo antes

31. Si se le diera la oportunidad, mi hijo/a estaría siempre tomando algo de beber
32. Mi hijo/a está interesado en probar alimentos que no ha probado antes
33. Mi hijo/a decide que no le gusta una comida, incluso sin haberla probado
34. Si se le diera la oportunidad, mi hijo/a estaría siempre comiendo algo
35. Mi hijo/a come cada vez más lento durante el transcurso de las comidas

Supplementary material 1: Exploratory Factor Analysis

Scale	Factor loading
**Food fussiness**	
7. My child refuses new foods at first	.816
10. My child enjoys tasting new foods	.949
16. My child enjoys a wide variety of foods	.554
24. My child is difficult to please with meals	.485
32. My child is interested in tasting food s/he hasn’t tasted before	.954
33. My child decides that s/he doesn’t like a food, even without tasting it	.576
**Food responsiveness**	
12. My child is always asking for food	.651
14. If allowed to, my child would eat too much	.639
19. Given the choice, my child would eat most of the time	.559
28. Even if my child is full up s/he finds room to eat their favourite food	.720
34. If given the chance, my child would always have food in his/her mouth	.729
**Satiety responsiveness**	
3. My child has a big appetite (-.331 in Factor 2)	.374
17. My child leaves food on his/her plate at the end of a meal	.632
21. My child gets full before his/her meal is finished	.695
26. My child gets full up easily	.793
30. My child cannot eat a meal if s/he has had a snack just before	.582
**Emotional undereating**	
9. My child eats less when angry	.827
11. My child eats less when s/he is tired	.667
23. My child eats more when she is happy	.729
25. My child eats less when upset	.820
**Enjoyment of food**	
1. My child loves food	.576
5. My child is interested in food (-.443 in Factor 1)	.369
20. My child looks forward to mealtimes	.492
22. My child enjoys eating	.578
**Desire to drink**	
6. My child is always asking for a drink	.698
29. If given the chance, my child would drink continuously throughout the day	.923
31. If given the chance, my child would always be having a drink	.927
**Slowness in eating**	
4. My child finishes his/her meal quickly	.694
8. My child eats slowly	.732
18. My child takes more than 30 minutes to finish a meal	.671
35. My child eats more and more slowly during the course of a meal	.612
**Emotional overeating**	
2. My child eats more when worried	.677
13. My child eats more when annoyed	.786
15. My child eats more when anxious	.806
27. My child eats more when s/he has nothing else to do (.533 in Factor 2)	.357
